# Clinical significance of the neutrophil-to-lymphocyte ratio in oligometastatic breast cancer

**DOI:** 10.1007/s10549-022-06726-w

**Published:** 2022-09-24

**Authors:** Yuka Inoue, Makoto Fujishima, Makiko Ono, Jun Masuda, Yukinori Ozaki, Tetsuyo Maeda, Natsue Uehiro, Yoko Takahashi, Takayuki Kobayashi, Takehiko Sakai, Tomo Osako, Takayuki Ueno, Shinji Ohno

**Affiliations:** 1 Breast Surgical Oncology, Cancer Institute Hospital of Japanese Foundation for Cancer Research, 3-8-31 Ariake, Koto-ku, Tokyo, 135-8550 Japan; 2 Department of Surgery, Kuma Hospital, Shinkokai Medical Corporation, 8-2-35, Shimoyamate-dori, Chuo-ku, Kobe-shi, Hyogo-ken 650-0011 Japan; 3 Medical Oncology, The Cancer Institute Hospital of Japanese Foundation for Cancer Research, 3-8-31 Ariake, Koto-ku, Tokyo, 135-8550 Japan; 4 Breast Medical Oncology, The Cancer Institute Hospital of Japanese Foundation for Cancer Research, 3-8-31 Ariake, Koto-ku, Tokyo, 135-8550 Japan; 5 Division of Pathology, The Cancer Institute of Japanese Foundation for Cancer Research, 3-8-31 Ariake, Koto-ku, Tokyo, 135-8550 Japan; 6 Breast Oncology Center, The Cancer Institute Hospital of Japanese Foundation for Cancer Research, 3-8-31 Ariake, Koto-ku, Tokyo, 135-8550 Japan

**Keywords:** Oligometastatic breast cancer, Neutrophil-to-lymphocyte ratio, NLR, Oligometastatic disease, Breast cancer

## Abstract

**Purpose:**

This study investigated the clinical impact of pretreatment neutrophil-to-lymphocyte ratio (NLR) on survival in patients with oligometastatic breast cancer.

**Patients and methods:**

We collected data from 397 patients who underwent primary breast surgery from 2004 to 2015 and developed recurrence during the follow-up. We reviewed the images and clinical information and defined OMD according to the European Society for Medical Oncology advanced breast cancer guidelines. The NLR was calculated using pretreatment data of primary breast cancer. The cutoff value of the NLR was determined by receiver operating characteristic curve with Youden Index.

**Results:**

Among 397 patients, 131 had OMD at recurrence. The low-NLR group included patients of significantly older age at primary cancer than those in the high-NLR group. A low NLR indicated a better overall survival (*p* = 0.023) after adjusting for relevant factors, including estrogen receptor status, surgical resection of metastatic disease, metastatic organ number, disease-free interval, and liver metastasis than did the high-NLR group. We developed prognostic models for OMD using six independent prognostic factors, including the NLR. The number of factors was associated with overall survival; patients with all six favorable factors showed a good overall survival of 90.9% at 8 years and those with four or more factors showed 70.4%.

**Conclusions:**

The NLR was an independent prognostic factor for overall survival in OMD. The number of favorable prognostic factors was associated with overall survival. A prognostic model, including the NLR, will help identify patients with a favorable prognosis.

**Supplementary Information:**

The online version contains supplementary material available at 10.1007/s10549-022-06726-w.

## Introduction

Because metastatic breast cancer (MBC) is an incurable disease, systemic therapy for palliation is the standard of care for affected patients [1, 2]. Approximately 30%–40% of patients with MBC will develop widespread metastases [3–5]. Although the advancement of systemic therapy for MBC has led to improved overall survival (OS), the median OS ranges widely from 8 months to 4 years [6–8]. However, in clinical practice, some patients achieve the status of no evident disease or long-term survival with controlled MBC.

Hellman and Weichselbaum first proposed the concept of oligometastatic disease (OMD) as a distinct cancer state between locally confined and systemically metastatic disease in 1995 [9]. Although patients with OMD are considered as potentially curable [6], there are no clear diagnostic criteria or treatment guidelines for OMD, and it is unclear who will benefit from treatment of curative intent. The European Society for Medical Oncology (ESMO) advanced breast cancer (ABC) guidelines define OMD as low volume metastatic disease with a limited number and size of metastatic lesions (up to five lesions and not necessarily in the same organ) [10]. Conversely, German experts define it as a limited number of metastases in one body organ [11].

Recent reports have shown that the neutrophil-to-lymphocyte ratio (NLR) correlates with survival in patients with cancer, including breast cancer [12, 13]. Although inflammatory cells and mediators in the tumor microenvironment play an important role in cancer progression, which may be reflected by systemic immune status [14], the clinical significance of NLR in oligometastatic breast cancer requires elucidation. This study investigated the clinical impact of pretreatment NLR on the survival of patients with oligometastatic breast cancer to help identify patients who will benefit from treatment of curative intent.

## Patients and methods

We analyzed the data from 397 patients who underwent primary breast surgery from 2004 to 2015 and developed recurrence in sites other than central nervous system during follow-up in our institution. Their (neo)adjuvant therapy was administered based on the guidelines of the Japanese Breast Cancer Society [15]. We reviewed the images and clinical information and defined OMD according to the ESMO ABC guidelines [10]. The NLR was defined as the absolute blood neutrophil count divided by the absolute lymphocyte count in the peripheral blood and was calculated using pretreatment data of the patients at the time of primary breast cancer. The cutoff value of the NLR was determined by receiver operating characteristics curve analysis using the Youden index. OS was defined as the period from the day of diagnosis of breast cancer recurrence until the day of death from any cause. JMP software, version 8.0.0 (SAS Institute, Cary, NC), was used for all statistical analyses. The results are expressed as the mean ± standard deviation or number. Group differences in continuous variables were assessed by the Mann–Whitney test. Group differences in categorical variables were assessed by the chi-squared test. Survival curves were plotted by the Kaplan–Meier method and compared by the log-rank test. Survival data were evaluated using a multivariate Cox proportional hazards model. Differences were considered significant at two-sided *p* < 0.05. A prognostic model for OMD was developed using favorable prognostic factors on multivariate analysis. Harrell's C-index was used to evaluate the prognostic strength of models [16].

## Results

Among 397 cases with recurrent breast cancer, 131 cases (33%) had OMD. The median follow-up from recurrence was 59 months (range 6–151 months). The cutoff value of the NLR was 2.52 by the Youden index using data from patients with OMD, and 93 patients (71%) were classified in the low-NLR group. The absolute counts of neutrophil and lymphocyte were plotted on a graph (Supplementary Fig. 1). Table 1 shows the background characteristics of patients at primary breast cancer according to the NLR groups. The low-NLR group included patients of significantly older age at primary breast cancer than those in the high-NLR group (*p* = 0.0026). There were no significant differences in clinical stage or subtype between the two groups. More patients received endocrine therapy as adjuvant therapy in the high-NLR group than in the low-NLR group (*p* = 0.0447).

**Table 1:** The background characteristics of patients with primary breast cancer according to NLR group

	High-NLR *n* = 38		Low-NLR *n* = 93		*P* value
Age at primary breast cancer (median, range)	47 (29–73)		55 (27–86)		**0.0026**
cStage					
0	0	(0)	3	(3)	0.40
I	6	(16)	18	(19)	
II	18	(47)	49	(53)	
III	14	(37)	23	(25)	
ER					
−	6	(16)	31	(33)	0.054
+	32	(84)	62	(67)	
PR					
−	18	(47)	50	(54)	0.56
+	20	(53)	43	(46)	
HER2					
−	31	(82)	72	(77)	0.65
+	7	(18)	23	(21)	
Subtype					
HR+HER2−	28	(74)	55	(59)	0.16
HR+HER2+	5	(13)	8	(9)	
HR−HER2+	2	(5)	12	(13)	
HR−HER2−	3	(8)	18	(19)	
Adjuvant endocrine therapy					
No	8	(21)	37	(40)	0.0447
Yes	30	(79)	56	(60)	
(Neo)adjuvant chemotherapy					
No	7	(18)	24	(26)	0.50
Yes	31	(82)	69	(74)	
(Neo)adjuvant anti-HER2 therapy					
No	35	(92)	78	(84)	0.27
Yes	3	(8)	15	(16)	
		(%)		(%)	

*NLR* Neutrophil-to-lymphocyte ratio, *ER* estrogen receptor, *PR* progesterone receptor, *HER2* human epidermal growth factor receptor type2, Statistically significant P values are shown in bold.

Table 2 shows the clinical characteristics of patients at recurrence and the treatment for OMD by the NLR groups. The low-NLR group included patients of significantly older age at recurrence than those in the high-NLR group (*p* = 0.0037). There were no significant differences in disease-free interval (DFI), number of metastatic organs, number of metastatic lesions, presence/absence of distant metastasis, or site of metastasis between the two groups. Moreover, there were no significant differences in the 1st-line therapy for OMD after recurrence between the two groups (Table 2).

**Table 2:** The clinical characteristics at recurrence and the treatment for OMD according to NLR groups

	High-NLR *n* = 38		Low-NLR *n* = 93		*P* value
Age at recurrence(median, range)	49 (30–78)		57 (31–86)		**0.0037**
DFI(month, median, range)	30 (6–97)		30 (2–98)		0.60
Number of metastatic organs					
1	36	(95)	83	(89)	0.57
2	2	(5)	9	(10)	
3	0	(0)	1	(1)	
Number of metastatic lesions					
1	20	(53)	57	(62)	0.73
2	7	(18)	16	(17)	
3	7	(18)	13	(14)	
4	3	(8)	3	(3)	
5	1	(3)	3	(3)	
Distant metastasis					1.00
Yes	25	(66)	62	(67)	
No	13	(34)	31	(33)	
Site of metastasis					
Local	4	(11)	13	(14)	0.77
Region LN	11	(29)	23	(25)	0.66
Bone	12	(32)	27	(29)	0.83
Liver	7	(18)	16	(17)	1.00
Lung/pleura	5	(13)	19	(20)	0.46
Distant LN	1	(3)	6	(6)	0.67
Surgery for OMD					
No	29	(76)	63	(71)	0.66
Yes	9	(24)	26	(29)	
RT for OMD					
No	32	(84)	75	(81)	0.80
Yes	6	(16)	18	(19)	
1st line					
Chemotherapy	11	(30)	44	(47)	0.079
Endocrine therapy	24	(65)	45	(48)	0.11
Anti-HER2 therapy*	5	(13)	18	(19)	0.61
		(%)		(%)	

*OMD* oligometastatic disease, *DFI* disease-free interval, *RT* radiotherapy, Statistically significant P values are shown in bold.

*A breakdown of the anti-HER2 therapy is shown in supplementary Table 1

**Fig. 1:**
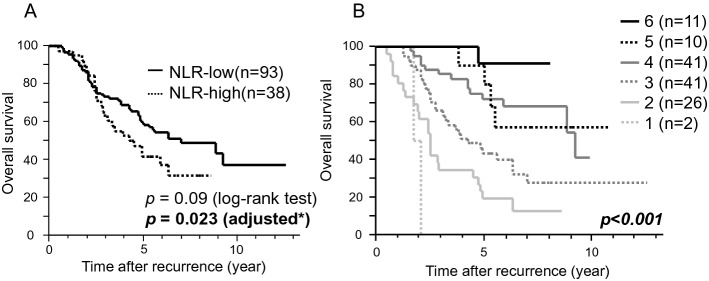
**A** Kaplan–Meier survival curves for overall survival in patients with OMD by NLR. A low NLR indicated better survival (*p* = 0.023) after adjusting for estrogen receptor status, age at recurrence, (neo) adjuvant chemotherapy, surgical resection of metastatic disease, metastatic organ number, DFI, and liver metastasis, than did a high NLR. *adjusted for estrogen receptor status, age at recurrence, (neo) adjuvant chemotherapy, surgical resection of metastatic disease, metastatic organ number, DFI, and liver metastasis. **B** Kaplan–Meier survival curves for overall survival in patients with OMD by the number of prognostic factors for OMD. Survival curves were drawn according to the number of the favorable prognostic factors, including no (neo)adjuvant chemotherapy, surgical resection, single metastatic organ, DFI > 2 years, no liver metastasis, and low NLR. The number of the factors was associated with post-recurrence survival. *OMD* oligometastatic disease, *DFI* disease-free survival, *NLR* neutrophil-to-lymphocyte ratio

Figure 1A shows the OS of patients with OMD by the NLR groups. Those in the low NLR had better OS (*p* = 0.023) after adjusting for estrogen receptor (ER) status, age at recurrence, (neo)adjuvant chemotherapy, surgical resection of metastatic disease, metastatic organ number, DFI, and liver metastasis (Table 3). In addition, no (neo)adjuvant chemotherapy (*p* = 0.0044), surgical resection (*p* = 0.0416), single metastatic organ (*p* = 0.05), DFI > 2 years (*p* = 0.007), and no liver metastasis (*p* = 0.02) were independent favorable prognostic factors (Table 3). In the whole population of patients with recurrent breast cancer, the NLR was prognostic (*p* = 0.0052) while it was not in patients with recurrent breast cancer without OMD (*p* = 0.134) (Supplementary Fig. 2A and B). Thus, the prognostic significance of NLR was more prominent for patients with OMD than for those without.

**Table 3:** Multivariate analysis for the overall survival

Variable		Multivariate analysis	
		HR (95%CI)	*P* value
ER	Positive vs. negative	0.91 (0.52–1.62)	0.74
Age at recurrence	< 50 vs. 50-	0.59 (0.33–1.08)	0.09
(neo) adjuvant chemotherapy	No vs. yes	0.38 (0.17–0.75)	**0.0044**
Surgical resection	Yes vs. no	0.53 (0.27–0.98)	**0.0416**
Number of metastatic organs	1 vs. 2 or more	0.45 (0.23–1.00)	**0.05**
DFI (year)	2- vs. < 2	0.40 (0.23–0.70)	**0.007**
Liver metastasis	No vs. yes	0.50 (0.29–0.89)	**0.02**
**NLR**	Low vs. High	0.52 (0.30–0.91)	**0.023**

*ER* estrogen receptor, *CI* confidence interval, *DFI* disease-free interval, *NLR* neutrophil-to-lymphocyte ratio, Statistically significant P values are shown in bold.

**Fig. 2:**
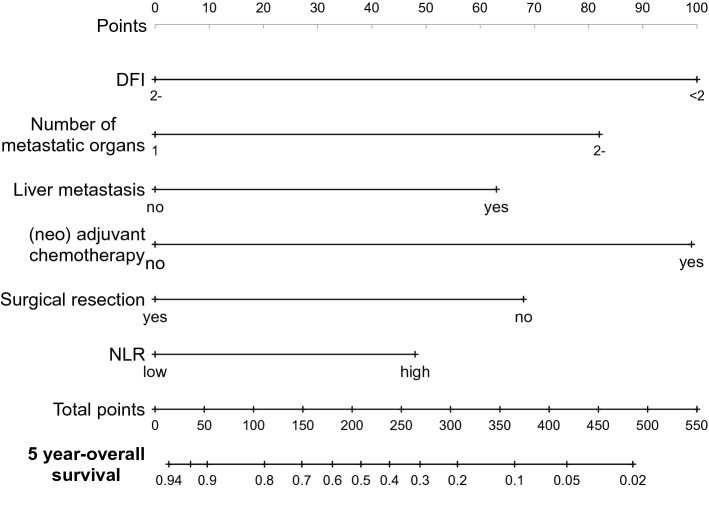
Nomogram for prediction of 5-year overall survival. A nomogram was constructed using the prognostic factors included in our model to predict 5-year overall survival. *DFI* disease-free survival, *NLR* neutrophil-to-lymphocyte ratio

To select patients with favorable prognosis likely to benefit from treatment of curative intent, prognosis was compared according to the number of favorable prognostic factors, including no (neo)adjuvant chemotherapy, surgical resection, single metastatic organ, DFI > 2 years, no liver metastasis, and low NLR. The number of prognostic factors was associated with OS (*p* < 0.001) (Fig. 1B). The OS at 8 years was calculated based on the number of included factors (Table 4). Patients with all six of the favorable factors showed an excellent 8-year OS of 90.9% (*p* = 0.001; hazard ratio [HR] = 9.14), while patients with five or more factors showed an OS of 75.6% (*p* = 0.0025) and those with four or more factors showed an OS of 70.4% (*p* < 0.001) (Supplementary Fig. 3). The concordance of each model was evaluated using Harrell's C-index, with a C-index of 0.737 for the model with NLR, which was higher than for the model without NLR (a C-index of 0.72). For the prediction of 5-year OS, we constructed a nomogram using these predictive factors (Fig. 2).

**Table 4:** The association the number of the prognostic factors and overall survival

Number of favorable prognostic factors	OS at 8 years (%)	HR	*P* value
6	90.9	9.14	0.001
≥ 5	75.6	3.68	0.0025
≥ 4	70.4	3.91	< 0.001

*OS* overall survival

## Discussion

This study showed that a low NLR was an independent favorable prognostic factor for breast OMD. Although the association between the NLR and prognosis in breast cancer has been reported in multiple studies [12, 13], to the best of our knowledge, this is the first report to show the prognostic significance of the NLR in OMD. A recent systematic review of breast OMD demonstrated that comparing to those without, patients with solitary metastasis, > 24-month interval between primary tumor and OMD, no or limited involved axillary lymph nodes at primary diagnosis, and hormone-receptor positivity were associated with better prognosis [17]. However, the NLR was not considered in that study. Our study results may enable clinicians to better predict the prognosis of patients with OMD by considering the NLR in the prognostic model.

Some studies have suggested that local treatment for metastatic lesions, such as surgery and radiotherapy, improves the survival of patients with OMD [18] The SABR-COMET trial, a phase 2 randomized trial, demonstrated that stereotactic ablative radiotherapy improved the prognosis of patients with OMD from different primary cancers, including breast cancer [19]. The SABR-COMET-3 trial, a phase 3 trial of the same concept, including breast cancer patients with OMD, is currently underway [20]. Some studies have examined outcomes after surgical resection of lung, liver, and brain metastases and suggested good long-term disease control and survival for selected patients [21–24]. There are also some case–control studies suggesting a survival benefit from surgical resection of metastatic lesions in patients with breast OMD [25–30]. However, these studies are retrospective, limited by number of patients, and conducted in highly selective cohorts; thus, a selection bias cannot be avoided. Indeed, the Korean case–control study showed better survival in patients with surgical resection of pulmonary metastases than in patients without surgery, but in the multivariate analysis, surgical resection did not remain an independent prognostic factor [26]. Therefore, it is unclear whether surgery itself contributes to the improved prognosis of patients with OMD. However, these studies do not exclude the possibility that surgical resection of metastatic lesions may provide some survival benefit in highly selected patients with favorable prognosis. A refined prognostic model that can select these patients with favorable prognosis would help to indicate those who would benefit from intensive treatment of curative intent, including surgery. Our results suggest that the addition of the NLR to conventional prognostic factors would be useful in such a prognostic model.

Most studies that examined prognostic factors in OMD focused on tumor-related factors, such as the number of metastatic lesions, metastatic organs, and tumor subtypes. However, it is now clear that host-related factors also affect patient prognosis. In this study, we showed that the NLR at primary diagnosis indicated the survival in patients with breast OMD, probably because it may reflect the host anti-cancer immune status.

### Limitations

One of the major limitations in this study was its small number of patients, which resulted partly from it being a single institutional study. Therefore, the survival analysis of this population needs to be interpreted with caution. We are planning a multicenter study with a larger population to confirm the results of this study. Another limitation is that the NLR at the time of recurrence could not be calculated because white blood cell fractions were not measured in all patients at recurrence. However, our result suggested that the NLR at primary cancer impacted survival even after recurrence, indicating the importance of the primary immune status throughout the disease course. The difference in the proportion of patients given adjuvant endocrine therapy between the two groups is another limitation (Table 1). Because female hormones have been reported to affect T-cell proliferation and neutrophil counts [31, 32], adjuvant endocrine therapy may have affected the systemic immune status. To reduce such a bias, we included ER status, which was associated with administration of adjuvant endocrine therapy, in the multivariate analysis, which showed that the NLR was prognostic independent of ER status (Table 3).

## Conclusion

The NLR was an independent prognostic factor for OS in OMD. The number of favorable prognostic factors was associated with survival. We developed a new prognostic model for OMD using the NLR, which will help to decide treatment strategy for patients with OMD.

## Supplementary Information

Below is the link to the electronic supplementary material.

Supplementary file1 (DOCX 12 kb)

Supplementary file2 (DOCX 105 kb)

## Data Availability

The data that support the findings of this study are available from the corresponding author upon reasonable request.
