# Supplementary material for: Clinical significance of the neutrophil-to-lymphocyte ratio in oligometastatic breast cancer
Source: Breast Cancer Res Treat. 2022 Sep 24;196(2):341–8. doi: 10.1007/s10549-022-06726-w (PMC9581831; doi:10.1007/s10549-022-06726-w)
Supplement: Supplementary file 1 — Supplementary file1 (DOCX 12 kb) [file 10549_2022_6726_MOESM1_ESM.docx]

**Supplementary Figure 1**

**The absolute neutrophil and lymphocyte counts in each patient**

**Supplementary Figure 2**

**Kaplan–Meier survival curves for overall survival in all patients (A) and in patients without OMD (B)**

*The adjustment was made for estrogen receptor status, age at recurrence, (neo) adjuvant chemotherapy, surgical resection of metastatic disease, metastatic organ number, DFI, and liver metastasis as in Figure 1A.

OMD: oligometastatic disease, NLR: neutrophil-to-lymphocyte ratio

**Supplementary Figure 3**

**Kaplan–Meier survival curves for overall survival in patients with OMD by different prognostic models**

Patients with all six favorable factors showed a good overall survival of 90.9% at 8 years, while patients with five or more factors showed an overall survival of 75.6% (p = 0.0025) and those with four or more factors showed an overall survival of 70.4% (p < 0.001).
