# Supplementary material for: Clinical significance of the neutrophil-to-lymphocyte ratio in oligometastatic breast cancer
Source: Breast Cancer Res Treat. 2022 Sep 24;196(2):341–8. doi: 10.1007/s10549-022-06726-w (PMC9581831; doi:10.1007/s10549-022-06726-w)

Supplementary Figure 1

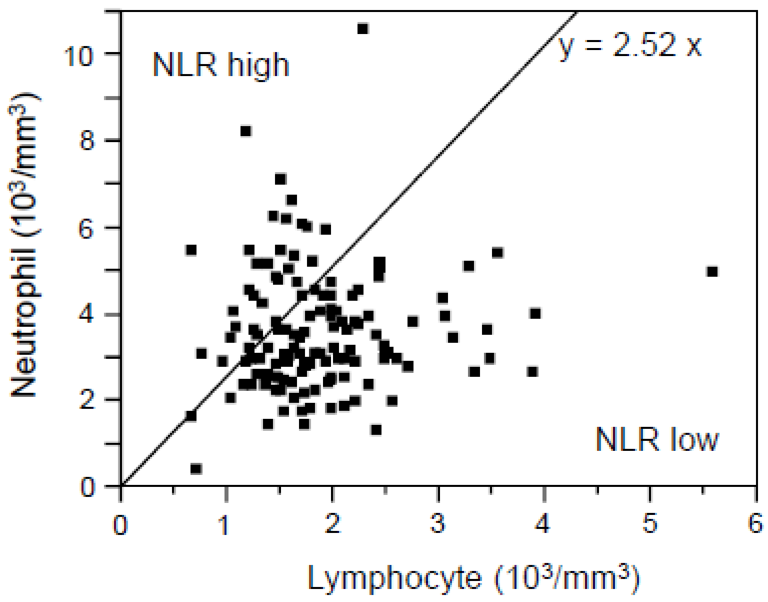

Supplementary Figure 2

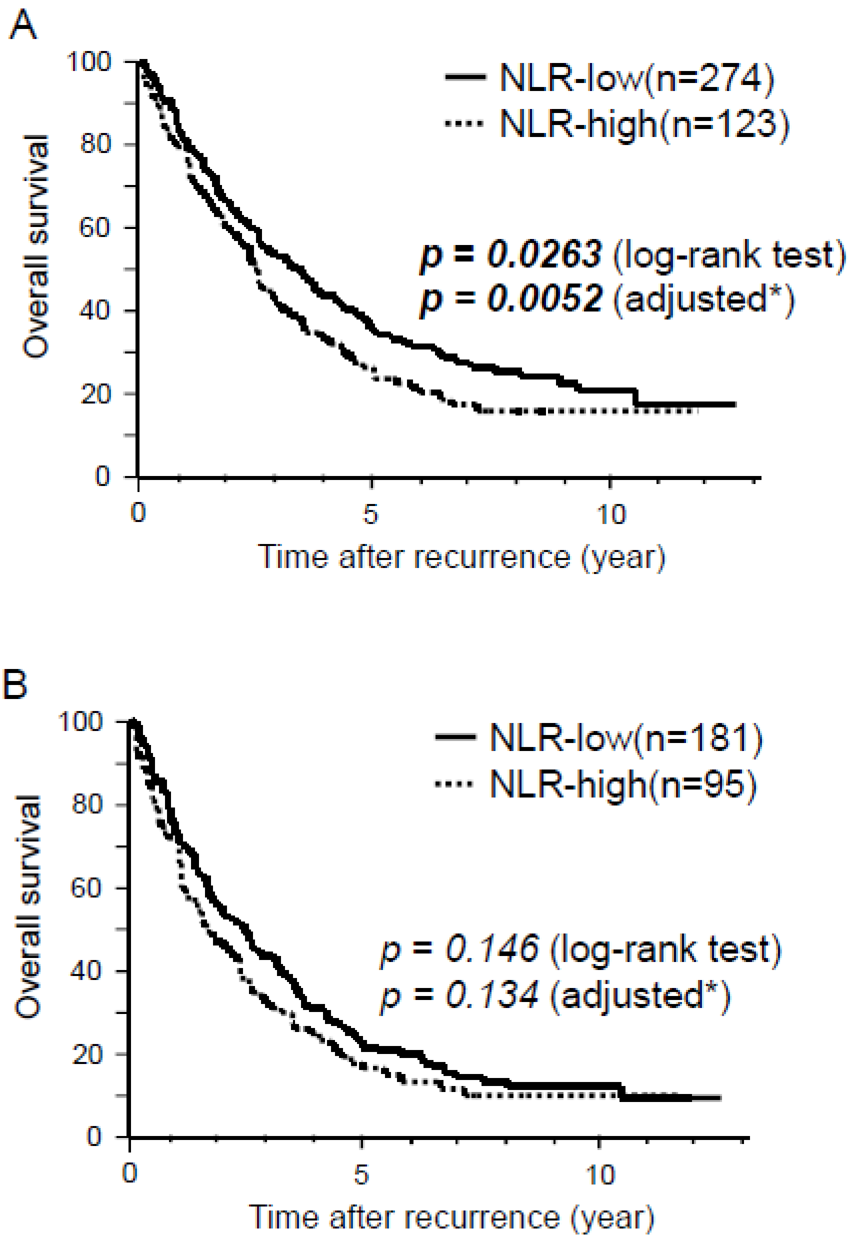

Supplementary Figure 3

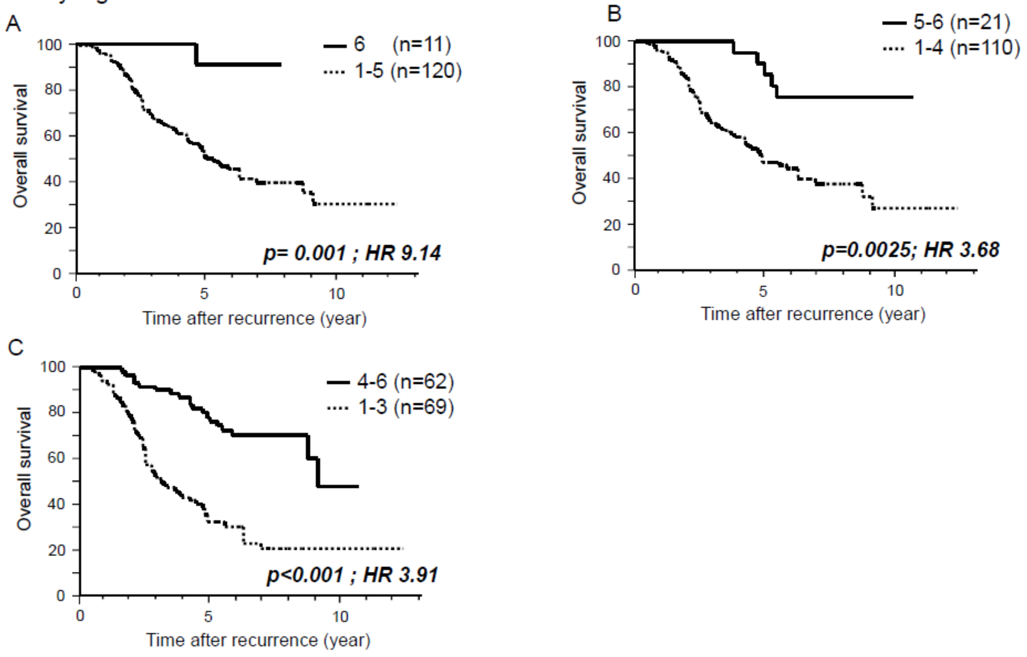

Supplement: Supplementary file 2 [file 10549_2022_6726_MOESM2_ESM.docx]
